# Intra-articular Administration of Allogeneic Adipose Derived MSCs Reduces Pain and Lameness in Dogs With Hip Osteoarthritis: A Double Blinded, Randomized, Placebo Controlled Pilot Study

**DOI:** 10.3389/fvets.2020.00570

**Published:** 2020-08-31

**Authors:** Chad B. Maki, Anthony Beck, Chan-Benami Cheuk Chi Wallis, Justin Choo, Thomas Ramos, Raymond Tong, Dori L. Borjesson, Fariborz Izadyar

**Affiliations:** ^1^VetCell Therapeutics USA, Santa Ana, CA, United States; ^2^Doctors Beck and Stone Clinic, Discovery Bay, Hong Kong; ^3^Wellness Veterinary Hospital, Yuen Long, Hong Kong; ^4^VetCell Therapeutics Asia, Mong Kok, Hong Kong; ^5^Department of Pathology, Microbiology & Immunology, School of Veterinary Medicine, Veterinary Institute for Regenerative Cures, University of California, Davis, Davis, CA, United States

**Keywords:** allogeneic, adipose, MSC, canine, OA, pain, lameness, inflammation

## Abstract

This study was conducted to investigate the therapeutic effect of allogeneic adipose-derived MSCs on dogs with hip osteoarthritis (OA). Twenty dogs with bilateral osteoarthritis of the coxofemoral (hip) joint, diagnosed by a veterinarian through physical examination and radiographs were randomly allocated into four groups. Group 1 served as a placebo control and were injected with 0.9% sodium chloride (saline) (*n* = 4). Group 2 were injected with a single dose of 5 million MSCs (*n* = 5). Group 3 received a single dose of 25 million MSCs (*n* = 6) and Group 4 received a single dose of 50 million MSCs (*n* = 5). Intra-articular administration of allogeneic MSCs into multiple joints did not result in any serious adverse events. The average lameness score of the dogs in the placebo control group (−0.31) did not show improvement after 90 days of intra-articular saline administration. However, the average lameness score of the all MSC-treated dogs was improved 2.11 grade at this time point (*P* < 0.001). Overall, sixty five percent (65%) of the dogs that received various doses of MSCs showed improvement in lameness scores 90 days after intra-articular MSC administration. Our results showed that intra-articular administration of allogeneic adipose derived MSCs was well-tolerated and improved lameness scores and reduced pain in dogs associated with hip OA. All doses of MSCs were effective. Subsequent studies with more animals per group are needed to make a conclusion about the dose response. The improved lameness effect was present up to 90 days post-injection. Serum interleukin 10 was increased in a majority of the dogs that received MSCs and that also had improved lameness.

## Introduction

Osteoarthritis (OA) or degenerative joint disease (DJD) is the most common form of arthritis in dogs affecting a quarter of the population (1, 2). Majority of OA in dogs occurs secondarily to developmental orthopedic disease, such as cranial cruciate ligament disease, hip dysplasia (HD), elbow dysplasia (ED), osteochondritis dissecans (OCD), and patella dislocation. In a small subset of dogs, OA occurs with no obvious primary causes and can be related to other contributing factors such as genetics, age, obesity, gender, exercise, and diet. It generally takes years of wear and tear before clinical symptoms of OA manifests, but in young patients with predisposing conditions such as hip dysplasia (HD) or elbow dysplasia (ED), symptoms may manifest as early as 1–2 years old (3, 4). OA involves degeneration, fibrillation and loss of cartilage, inflammation, and hyperplasia of the synovial membrane, abnormal proliferation of bone (bone spurs—osteophyte and/or enthesophyte production) and eventually exposure of subchondral bone (3).

OA is an immensely important degenerative disease as it is progressive, debilitating, painful, and is associated with bone and cartilage changes and inevitably leads to joint failure. Most common medical management of OA includes systemic administration of anti-inflammatory drugs such as non-steroidal anti-inflammatories (NSAIDs), corticosteroids and intra-articular administration of Hyaluronic Acid (HA) that acts as lubricant (5). An increase in knowledge about the inflammatory and anti-inflammatory molecules involved in joint inflammation has led to developing more specific drugs for arthritis (6, 7). Recent studies revealed the Role of interleukin 1 (IL-1) and Interleukin 1 receptor antagonist (IL-1Ra) in joint inflammation and cartilage degradation (8). IRAP which is a soluble protein and binds and neutralizes IL-1, is shown to have anti-inflammatory properties and its utilization for treatment of OA in dogs and horses are reported (9, 10). Utilization of anti-inflammatory cytokines for reducing joint pain and inflammation is also reported. Among anti-inflammatory cytokines, it has been shown that IL4-10 fusion protein has chondroprotective, anti-inflammatory, and potentially analgesic effects in the treatment of osteoarthritis (11).

Cell-based therapies are being considered as potential disease modifying agents for the treatment of OA both in human (12, 13) and companion animals (3, 14, 15). Different studies showed that Platelet-rich plasma (PRP), through their secretory cytokines, have a positive effect on reducing inflammation and symptoms of OA (16, 17). However, due to the short life span of platelets *in vivo* (4–7 days), the effect seems to be transient. Mesenchymal stem cell (MSC) therapy for OA is intriguing for many reasons, but four properties in particular make them appealing: (1) they are a bioactive living system that can interact with the host immune system and maintain a lasting and adaptable effect; (2) they are anti-inflammatory and therefore should slow or stop the progress of OA and improve clinical symptoms, (3) they can “home” to the site of injury within the joint to have a more targeted effect, and (4) they have the potential to create new cartilage either directly by differentiation into chondrocytes or by paracrine function to recruit and activate nascent stem cell and progenitor cell populations (18–20).

MSCs can be obtained from different sources, such as bone marrow, periosteum, umbilical cord blood, dermis, muscle, infrapatellar fat pad, synovial membrane, and adipose tissue (20). Among these sources, adipose-derived mesenchymal stem cells (AD-MSCs) are attracting attention as an alternative to the better studied bone marrow mesenchymal stem cells (BM-MSCs) (21–25). The reasons for increased interest in AD-MSCs reside in their abundance (~5% of the nucleated cells in adipose vs. 0.0001–0.01% for the bone marrow), the ease with which they can be harvested (with the advantages of lower donor-site morbidity), and their rapid expansion and high proliferation potential (26, 27). Utilization of adipose-derived MSCs in treatment of articular cartilage has been repeatedly reported (26, 28). A positive effect of autologous adipose derived MSCs on alleviating symptoms of OA is reported in human (29–32), equine (33), and canine (34) patients.

The mechanism by which MSCs reduce pain and increase mobility in dogs with osteoarthritis is not completely understood. However, there is evidence indicating that MSCs interact with resident immune cells within the joint environment, secrete anti-inflammatory cytokines, and reduce inflammation in the arthritic joint (35, 36). Also, given the capacity of MSCs to differentiate toward the chondrogenic lineage, OA has been proposed as one of the primary areas for MSC-based therapy for cartilage regeneration. Thus, MSCs could be effective in treating OA by repairing the compromised tissues and replacing nascent cell loss (37).

Recently, the effect of allogeneic adipose derived MSCs for treatment of canine OA has been studied (38, 39), and it was concluded that intra-articular administration of MSCs is safe and effectively reduces lameness and increases mobility as compared to the control group. However, in these studies all the dogs received the same dose of MSCs and the effect of various doses was not investigated. Also, the mechanism by which MSCs exert their therapeutic effect on reducing inflammation in arthritic joints has not been investigated. Therefore, this randomized, double blinded, placebo controlled clinical trial was designed to better understand the effect of different doses of off-the-shelf allogeneic adipose MSCs and their possible mode of action on improving lameness and mobility in dogs with OA.

## Materials and Methods

### Donor Eligibility and Collection of Adipose Tissue

For this study, a healthy 5-month-old female dog was selected as a tissue donor. A licensed veterinarian performed a physical examination and collected intraabdominal fat during a routine ovariohysterectomy surgical procedure under general anesthesia. The patient was recovered as per normal procedure without any adverse events. Immediately following tissue extraction, the tissue was transferred to a sterile tissue collection container in sterile, cold phosphate buffer solution (PBS) and was sent to VetCell Therapeutics USA's (VCT-USA's) biomanufacturing facility in a validated shipper. The shipper maintains the temperature between 2 and 8°C for up to 24 h, and this was confirmed with a data logger.

### Manufacturing of MSCs

Upon arrival, the box containing adipose tissue was transferred to the VCT production facility. Adipose tissue processing was performed according to VCT-USA's Standard Operating Procedures (SOPs). Cells were quality control-tested for the number of cells isolated, viability, sterility, and environmental monitoring of the biosafety cabinet. Cell counts were performed with an automated cell count (Chemometec NC-200 Nucleocounter). Cells were then culture expanded through passage 2 (P2). After culturing, the MSCs were cryopreserved in Cryostor CS10 cryopreservation solution (BioLife Solutions) in a controlled-rate freezer (Planer). All MSC batches undergo quality control (QC) testing including confirmation of high cell viability and recovery, and tests for sterility via direct inoculation culture [Gram +, Gram (–), fungi, yeast], confirmation of <0.5 EU/mL endotoxin via the kinetic chromogenic Limulus Amebocyte Lysate (LAL) assay and polymerase chain reaction (PCR) for Mycoplasma at a commercial testing laboratory. A panel of surface markers are determined via flow cytometry (CD29^+^, CD90^+^, CD34^−^ MHC-II^−^, and CD45^−^) to confirm MSC identity and purity. A karyotype analysis was performed to assure lack of chromosomal abnormalities (data not shown). For this study, only one batch of MSCs was used. Additionally, this batch tested negative for 29 adventitious agents via PCR testing performed by a clinical veterinary diagnostic laboratory (data not shown). This batch also successfully differentiated to adipocytes, chondrocytes, and osteocytes as evidenced by positive Oil Red-O, Alcian Blue, and Alizarin Red S staining, respectively (data not shown). Furthermore, the MSCs were potent as determined by their ability to inhibit activated lymphocyte proliferation by 45% and secrete the immunomodulatory cytokines prostaglandin E_2_ (PGE_2_) and indoleamine 2,3-dioxygenase (IDO) in a mixed leukocyte reaction (MLR) assay (data not shown).

### MSC Storage and Shipment

After cryopreservation, the MSCs were transferred to a vapor-phase liquid nitrogen (LN) Dewar (Planer) at cryogenic temperature (<-150°C). The temperature of the Dewar was constantly monitored and recorded to ensure the MSCs remained at cryogenic temperature for the duration of storage. Cell transfer was performed in a validated LN vapor phase shipper that was also monitored to confirm proper temperature regulation. The VCT-Asia lab received the cells and transferred them to another Dewar. The MSCs remained in this condition until they were thawed for preparation of therapeutic dosing.

### Preparation of Therapeutic Doses of MSCs

Preparation of a therapeutic dose was performed in an ISO class 5 biosafety cabinet at VCT-Asia lab. The MSCs were thawed in a ThawStar automated cell thawing system (Asterbio). After thawing, the cells were quickly removed from the cryopreservation medium by dilution, centrifugation, and resuspension in a Dulbecco's PBS (DPBS)-fetal bovine serum (FBS) buffer. After counting, the appropriate cell dose was washed with DPBS, centrifuged again, and resuspended in 0.6 mL of 0.9% sodium chloride (saline). The cells were then loaded into a sterile, plastic 1 mL Leuer lock syringe, the air was expelled, and the syringe secured with a sterile cap and placed in a sterile syringe sleeve identifying the name and study ID number of the recipient. These syringes were then transferred directly into a cooled (2–8°C) shipping container and delivered to veterinary clinics no more than 4 h after being prepared.

### Patient Enrollment Criteria

#### Inclusion Criteria

Both the veterinarian and owner were required to sign and agree to the terms and risks associated with the study (double-blinded study, long term follow up, no other intervention, etc.). The patient was at least 1 year old and weighed at least 9 kg (19.8 lbs.). Any breed and either sex were acceptable. The patient must have been diagnosed with OA of one or both hip (coxofemoral) joints by a licensed veterinarian. Patients must have had noticeable lameness, limited range of motion, and evident pain on palpation/manipulation at the time of evaluation. Radiographs must have shown evidence of arthritic changes and patient had to have at least 1 month of symptoms associated with OA and must have undergone at least 1 month of medical and/or physical therapy/cage rest management with little or no improvement. Patient must have had a body condition score (BCS) of 7/9 or less and had to maintain a consistent weight throughout the study. The patient must have had a minimum of 1 week of no treatment with non-steroidal anti-inflammatories (NSAIDS) prior to the study and no NSAID dosing was allowed throughout the study. Any additional treatments had to be ceased a minimum of 1 week prior to the start of the study. No additional treatments were allowed during the study including, but not limited to, Adequan injections, other joint injections, other pain or anti-inflammatory medications, physical therapy, acupuncture, low level laser, etc. Tramadol use was acceptable for 2–3 days post-arthrocentesis to help alleviate acute pain if needed, but was not allowed past 3 days post-arthrocentesis. Institutional Animal Care and Use Committee (IACUC) approval was not required because this study was conducted at private veterinary clinics using client owned dogs with owner consent. Also, approval was not required for this type of study under local legislation at the time. The dogs enrolled in this study were maintained in the owner's residence and were brought to the investigator sites for treatment and evaluation at the study's defined time points.

#### Exclusion Criteria

Must not have any additional known significant illness, infection or disease or recent surgery on affected area. If surgery was performed previously, it must have been at least 1 year since surgery. If a total hip replacement procedure had been performed, this joint could not be used for the study, but the contralateral joint could have been used.

### Administration of Cells Into Coxofemoral Joints

Some patients were premedicated with acepromazine and butorphanol, and all patients were placed under general anesthesia by propofol induction, intubated and maintained on a mixture of isofluorane and oxygen. Patients were positioned in lateral recumbency, with the joint of interest dorsal (facing up). The dorsal hind limb was kept in a normal standing anatomical position and allowed to hang off the edge of the surgery table to help expose the joint space. The injection site was prepared as for aseptic surgical procedure by clipping the hair and scrubbing with chlorhexidine surgical scrub, followed by a final alcohol scrub. The surgeon was sterile scrubbed, gowned and gloved. A 22-gauge spinal needle was directed perpendicular to the skin, just dorsal to the greater trochanter and into the coxofemoral joint space. Commonly, external rotation and distal traction was applied to the limb to aid entry into the coxofemoral joint space. Entry into the joint space was confirmed by “surgeon-feel” of a slight “pop” through the joint capsule and/or by aspiration of synovial fluid with a Leuer lock syringe. In some patients, no synovial fluid was aspirated. The injection syringe was inverted at least 10 times to gently mix the MSCs. Keeping the needle in its place within the joint, the injection syringe was securely attached to the hub of the needle. The cells were slowly injected within the joint space over 5 s. The injection syringe and needle were then withdrawn from the patient and firm pressure was applied over the puncture site for 30 s, followed by taking the limb through a full range of motion a few times. This procedure was repeated on the contralateral coxofemoral joint.

### Patient At-home Care Post- intra-articular Injection

Owners were instructed to keep the patient quiet at home. Patients were permitted to perform slow, well-controlled 5 to 10-min leash walks up to 2 times a day for the first 7 days post-injection. After this, owners were instructed to perform slow, well-controlled leash walks for no more than 30 min twice daily for the duration of the study. Owners were instructed not to allow any running, jumping, playing, stairs, or over-exertion. Patients were not allowed to start any additional rehabilitation programs and were instructed to maintain a fairly constant weight for the 3-month study duration. Over-exertion and any dog-dog interactive play or going up and down stairs and jumping on and off any furniture was avoided.

### Study Design

In total, 20 dogs were enrolled in two veterinary clinics in Hong Kong. Sixteen dogs were injected intra-articular (IA) with various doses of MSCs. Five dogs received 5 million (5M) cells per joint, six dogs received 25 million (25M) cells per joint and five dogs received 50 million (50M) cells per joint. Four dogs in the placebo group were injected IA with saline alone (Table 1).

**Table 1:** Distribution of patients enrolled in this study among different treatment groups.

**Treatment group**	**Patient ID number**	**Age (years)**	**Sex**	**Weight (kg)**	**Breed**	**Total cells × 10^**6**^**	**Joints injected**	**Limb discomfort post-injection (Y/N)**	**Lameness improved (Y/N)**
Placebo	P-1	8	F	21.5	German Shepherd	0	Hips	Y	Y
Placebo	P-2	10	F	46.1	Swiss Mountain Dog	0	Hips	N	N
Placebo	P-3	13	F	22.6	Mongrel	0	Hips	N	N
Placebo	P-4	7½	F	10.8	Bichon Frise	0	Hips	N	N
5M	5-1	11½	F	34	Golden Retriever	10	Hips	Y	N
5M	5-2	13	F	25.7	Labrador Retriever	10	Hips	N	Y
5M	5-3	14	M	21.9	Golden Retriever	20^**^	Hips Elbows	N	Y
5M	5-4	11	M	31.8	Mongrel	10	Hips	N	Y
5M	5-5	1	M	40	Bernese Mountain Dog	10	Hips	N	Y
25M	25-1	14	F	22.5	Labrador Retriever	50	Hips	Y	N
25M	25-2	11	F	29.5	Golden Retriever	50	Hips	N	N
25M	25-3	13	F	11.8	Cocker Spaniel	100^**^	Hips Stifles	N	Y
25M	25-4	11	M	43.6	Labrador Retriever	50	Hips	N	Y
25M	25-5	14	M	29.3	Golden Retriever	50	Hips	Y	Y
25M	25-6	4	M	25.3	Bulldog	50	Hips	N	N
50M	50-1	12	F	21	Mongrel	100	Hips	N	Y
50M	50-2	11 ½	M	29	Golden Retriever	100	Hips	N	N
50M	50-3	3	M	46.7	Caucasian Shepherd	150^**^	Hips R-Stifle	N	Y
50M	50-4^*^	4	F	23.3	Bulldog	100	Hips	Y	Y
50M	50-5^*^	14	F	23.7	Golden Retriever	100	Hips	N	N

* *Two dogs were excluded from the study because they received other medications or treatments throughout the study that were not permitted in the inclusion criteria*.

** *Dogs injected in more than 2 joints*.

*5M: 5 × 10^6^ cells*.

*25M: 25 × 10^6^ cells*.

*50M: 50 × 10^6^ cells*.

The study was randomized and double blinded. Each dog was assigned a number. Owner and veterinarian-1 (Vet-1) were not aware of what was injected. Veterinarian-2 (Vet-2) performed the IA injection procedure and knew what each patient received, and this information remained confidential to Vet-2 and VCT researchers only.

The following procedure was applied to all patients: A VCT-Asia technician supplied the treatment syringes in a shipping container on each surgery day. Each treatment was assigned randomly to denote treatment vs. placebo and this was documented for each patient by the VCT technicians and Vet-2 (surgeon). Vet-1 was the pre- and post-surgery consultant and diagnostician for each individual patient.

### Clinical and Paraclinical Examinations

Lameness scoring was performed by orthopedic examination with range of motion and pain assessment while standing, and lameness assessment at a walk and trot [(40, 41); Supplemental Table 1]. Pain and lameness score was performed prior to injection, Day 0 (day of injection), Day 5 after injection (no trot), and Day 30 and Day 90 after injection. Radiographs were taken by a licensed veterinarian only to confirm degenerative joint disease prior to injection. No radiographs were taken during or after the completion of study. Images were taken from three different views: ventro-dorsal extended, ventro-dorsal “frog leg” and right lateral views. Peripheral blood was taken prior to cell injection, at Day 0 (day of injection) and at Day 5, Day 30, and Day 90 after injection for anti-/pro-inflammatory and immunomodulatory biomarkers. Owner pre- and post-canine brief pain inventory score (CBPI) form was used to assess the owner's perception of the animal's pain and mobility at home at Day 0 (day of injection), and at Day 5, Day 30, and Day 90 after injection (Supplemental Table 2). According to the lameness scoring system, >2 points change on the scale was considered an improvement. In addition, veterinarian pre- and post-assessment forms were used to document patient lameness scores at Day 0 (day of injection), and at Day 5, Day 30, and Day 90 after injection (Supplemental Table 1).

### Cytokine Measurements in Canine Plasma

Serum levels of interleukin-1 receptor antagonist protein (IL-1RA) were measured using a Kingfisher Biotech ELISA kit (St. Paul, MN), following the manufacturer's recommendations as modified by Huggins et al. (42). Briefly, Nunc-Immuno MaxiSorp 96-well plates (Nalge Nunc, Rochester, NY) were coated with 100 μL of 2 μg/mL capture antibody. A standard curve ranging from 5,000 pg/mL to 19 ng/mL was prepared. Samples were diluted 1:8 with reagent diluent before plating, and 100 μL of standards and samples were run in duplicate. The detection antibody was diluted to 400 ng/mL, and 100 μL of this was added to each well. ELISAs were read at 450 nm, with 540 nm background subtraction, on a Synergy HT Multi-Mode microplate reader with Gen5 software (Biotek, Winooski, VT). Concentrations were calculated on a 4-parameter non-linear regression curve. Serum levels of interleukin-10 (IL-10) were measured with a canine cytokine magnetic bead panel (CCYTOMAG-90K, Millipore, Billerica, MA). Samples were diluted 1:2 with assay buffer and run according to the manufacturer's instructions. Standard curves from 50,000 to 12.2 pg/mL were prepared and run at the same time. Plates were read on a Luminex 200 instrument using Xponent software (Luminex Corporation, Austin, TX). Concentrations were calculated on a 5-parameter logistic curve.

### Statistical Analysis

Results were presented as mean ± standard deviation. For side by side comparison of groups, the data was analyzed with two sample *t*-test or Chi-Square and for analysis of variance ANOVA was used. *P* < 0.05 was considered statistically significant.

## Results

### Distribution of Sex, Age, and Weight of the Recipients in Different Groups

In total, 20 subjects were enrolled in this study. Two subjects were excluded because they received other medications or treatments during the study that were not permitted in the inclusion criteria. Information about the patient signalment is provided in Table 2.

**Table 2:** Sex, age, and weight of the dogs in each treatment group.

**Treatment group**	**Total number**	**Number of females (%)**	**Number of males (%)**	**Average age (years)**	**Average weight (kg)**
Placebo	4	4 (100)	0 (0)	9.6	25.2
5M	5	3 (60)	2 (40)	10.1	30.7
25M	6	3 (60)	2 (40)	11.1	27
50M	3	1 (33)	2(67)	8.8	32.2

*5M: 5 × 10^6^ cells*.

*25M: 25 × 10^6^ cells*.

*50M: 50 × 10^6^ cells*.

### Effect of Age, Sex, and Severity of Lameness on Therapeutic Response

**Figure 1:**
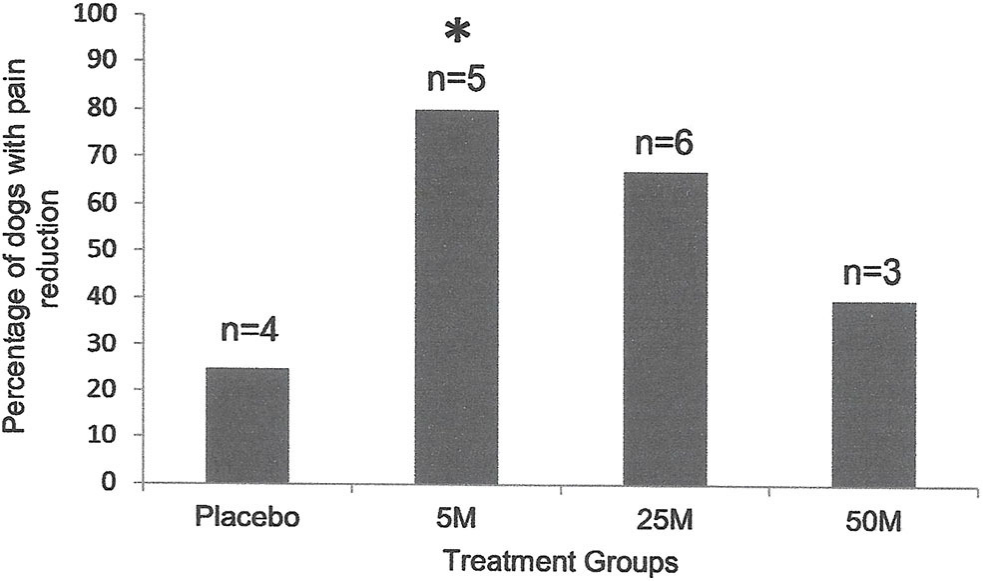
Percentage of the dogs with severity and interference of pain reduction in Placebo (*n* = 4) as compared with the dogs treated with 5 × 10^6^ (*n* = 5), 25 × 10^6^ (*n* = 6), or 50 × 10^6^ (*n* = 5) MSCs 90 days after injection. Pain severity or interference reduced with all doses of MSCs. However, only the dogs that received 5 million cells (*n* = 5) showed a significant reduction (*P < 0.01; Chi Square) as compared to the placebo group (*n* = 4).

**Figure 2:**
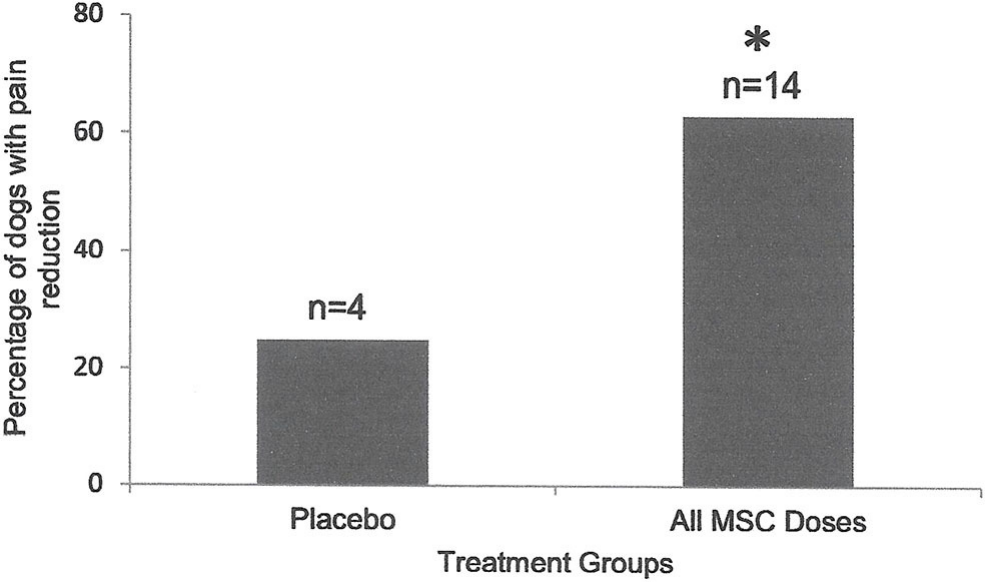
Percentage of the dogs with severity and interference of pain reduction in Placebo (*n* = 4) was compared to those in dogs that received various doses of MSCs combined (*n* = 16). Ninety days after intra-articular injection, only 25% of dogs (1/4) in the placebo group showed pain severity or interference reduction, while 64% of dogs (9/14) that received MSCs showed significant (*P < 0.001; Two Sample *t*-Test) reduction in pain severity or interference.

Sixty six percent (2/3) of the dogs under 10 years old had improved lameness following MSC administration. Similarly, seven of the 22 (63%) of dogs over 10 years old showed improved lameness after cell therapy (*P* = 0.65). Three of six (50%) female dogs treated with various doses of MSCs had improved lameness. Similarly, six of eight (75%) male dogs treated with MSCs showed lameness improvement which was not significant (*P* = 0.12). This indicates that sex of the recipients had no influence on the therapeutic effect of MSCs. Interestingly, 86% percent (6/7) of the dogs with low to moderate lameness (score 2–5) responded to MSC administration and showed improved lameness scores. However, only 3/4 (75%) of the dogs with severe lameness (score 6–10) responded to MSC administration and showed improved lameness scores (*P* < 0.05). Owner assessment evaluations (CBPI) also supported lameness scores (Figures 1, 2).

### Effect of IA MSC Administration on Lameness Score

**Figure 3:**
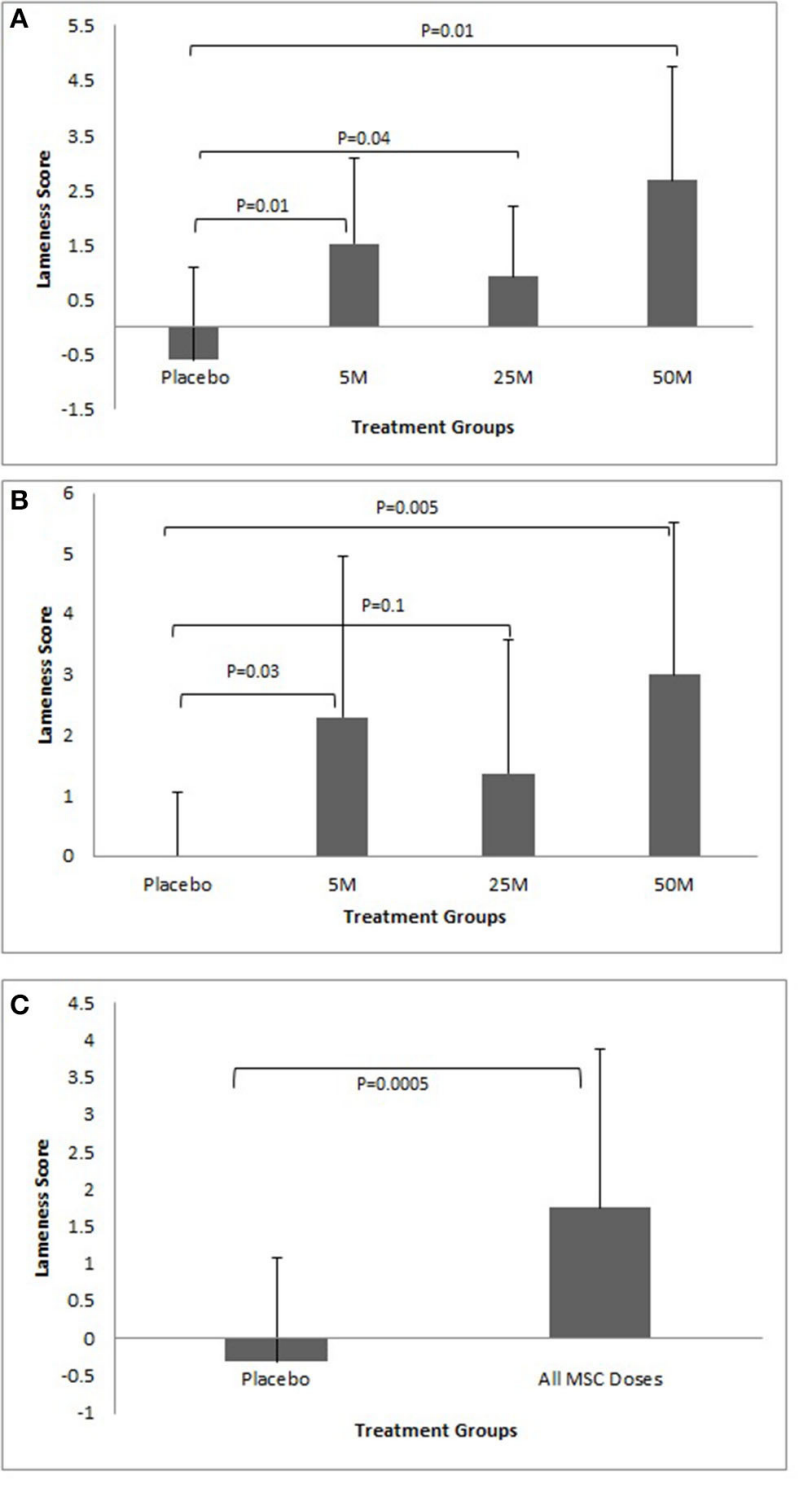
**(A)** Lameness improvement during the first 30 days. Note all doses of MSCs improved lameness. The response to MSC therapy was varied among different patients. Overall, there was a trend that higher doses of MSCs resulted in more improvement. No improvement in average lameness score was seen in the Placebo group. **(B)** Lameness improvement after 90 days showed similar results as after 30 days. All doses of MSCs improved lameness and the response to MSC therapy varied among different patients in all groups. Overall, there was a trend that higher doses of MSCs resulted in more improvement. No improvement in average lameness scores was seen in the Placebo group. **(C)** Lameness Improvement due to MSC administration. The graph represents cumulative data collected at days 30 and 90 regarding lameness improvement in Placebo group as compared to all MSC groups. The graph clearly shows a significant improvement in lameness score in the MSC groups as compared to the Placebo group (*P* < 0.001).

Dogs injected with 5, 25, and 50 million MSCs/joint showed significant improvement in their lameness scores during the first 30 days after injection (*P* < 0.05). The dogs in the placebo group did not show any lameness improvement during the first 30 days (Figure 3A). Dogs injected with 5 (*P* = 0.03) and 50 (*P* = 0.01) million MSCs/joint showed significant improvement in their lameness score after 90 days. Dogs injected with 25 million MSCs/joint also had improvement, but due to a large standard deviation, the difference was not significant (*P* = 0.1). Similar to the 30 day data point, the dogs in the placebo group did not show any lameness improvements after 90 days (Figure 3B). When all data from all MSC doses combined were compared against the placebo group, the results clearly showed that a single IA dose of MSCs significantly (*P* < 0.0001) improved lameness during the 90-day study period (Figure 3C). There were five patients in total that experienced increased pain per owner up to 5 days post-injections. They were one Placebo, one 5M, one 25M, and one 50M. Owner reported at least one of the following symptoms: pain, walking slower, increased limping. One veterinarian investigator noted resentment with joint manipulation with one 25M patient which was not noted at the 30 day follow up exam. No other adverse events occurred after intra-articular administration of MSCs.

### Serum Levels of IRAP and IL-10 as Biomarkers for Improved Lameness

**Figure 4:**
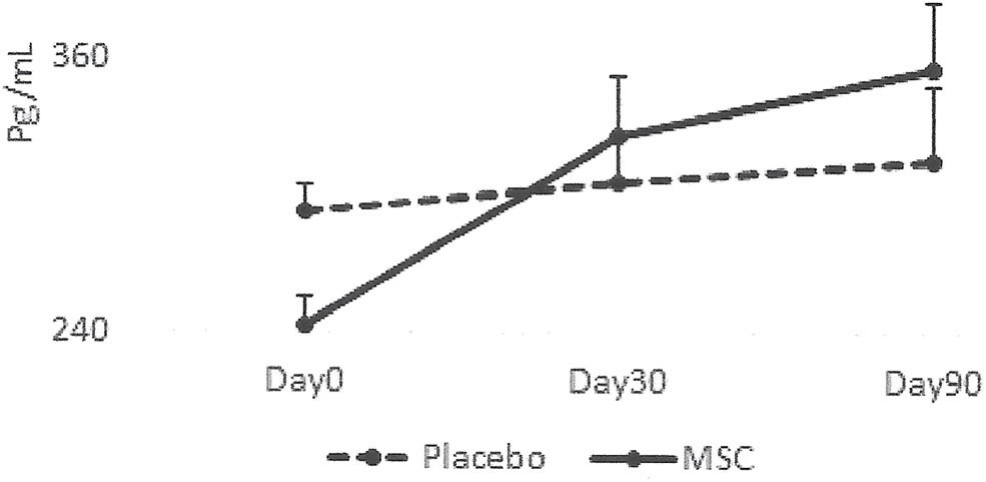
Serum levels of IRAP in patients with improved lameness score. This graph represents IRAP concentrations in the dogs with lameness improvement in the Placebo group (2/3) vs. the dogs with improved lameness score that received MSCs (6/7) during the study. IRAP concentrations did not change significantly (*P* = 0.9) in dogs with improved lameness score in the Placebo group (2/3). There was a trend showing that IRAP concentration increased in dogs with improved lameness following MSC administration (6/7) although the difference was not significant (*P* = 0.6).

**Figure 5:**
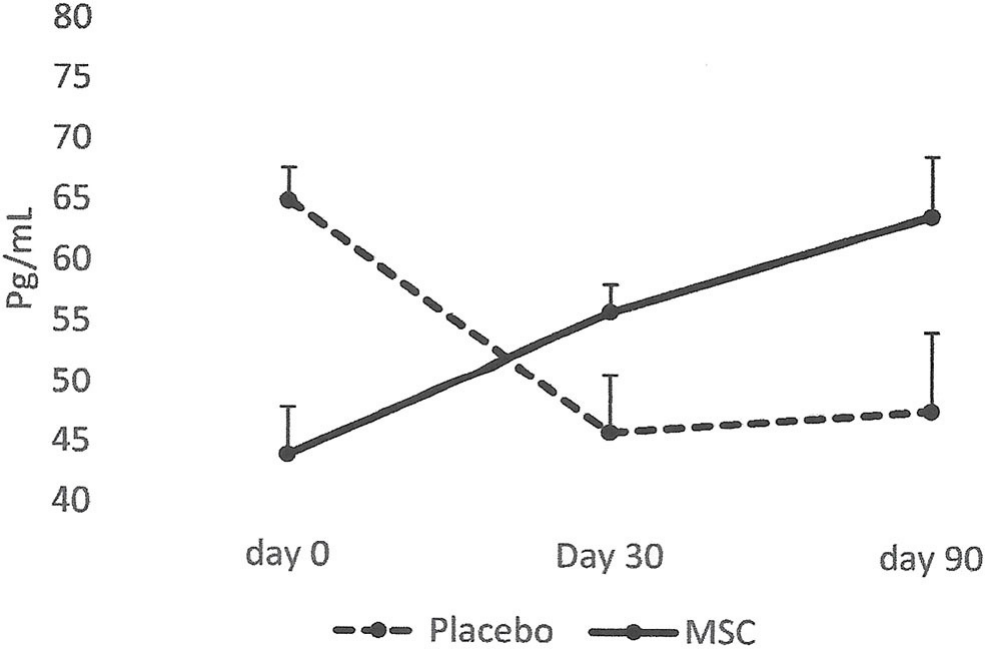
Serum levels of IL-10 in patients with improved lameness score. The data showed that the average level of IL-10 in serum increased only in the dogs treated with MSCs (4/6) and remained high at day 90. On the contrary, serum levels of IL-10 diminished in dogs in the Placebo group and remained low (2/2).

**Figure 6:**
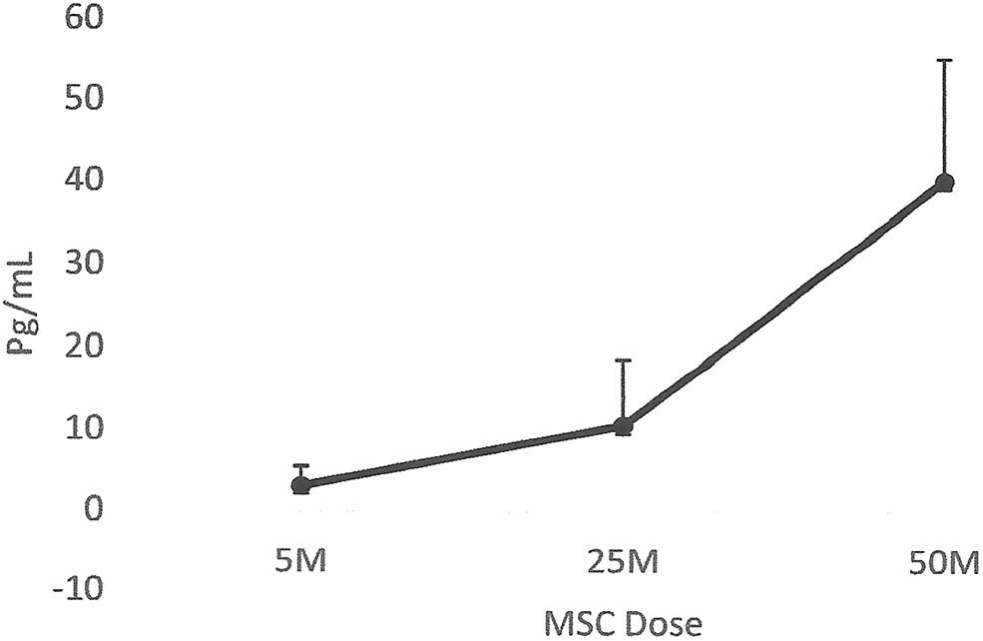
Correlation between serum IL-10 and MSC dose. The data showed that the level of IL-10 in peripheral blood 90 days after intra-articular administration of various doses of MSCs increased in a dose dependent manner. However, due to the small sample size, the difference was not significant (*P* = 0.06).

The results collected from this small group of dogs showed that all the dogs that had lameness improvements after 30 days, regardless of whether they were in the placebo or MSC-treated groups, had an elevated blood IRAP. This indicates that elevated blood IRAP 30 days after intra-articular injection is a good indication of improvement of lameness irrespective to the treatment group (Figure 4). The data also shows that, contrary to elevated IRAP, IL-10 was not elevated in the plasma of any placebo dogs that had improvement in lameness scores. However, 67 percent of dogs (4/6) with improved lameness that were treated with various doses of MSCs showed elevated serum IL-10. This indicates that an elevated level of IL-10 in peripheral blood, 30 days after MSC administration might be a good indicator of the improved lameness due to cell therapy (Figure 5). Based on this result, peripheral serum IL-10 levels might be a good indication of effectivity of MSC therapy for OA. Furthermore, our data showed that the level of IL-10 in peripheral blood 90 days after intra-articular administration of various doses of MSCs increased in a dose dependent manner (Figure 6). However, a variation in response and a small sample size may have contributed to make the difference not significant (*P* = 0.06).

## Discussion

Our double-blinded, placebo-controlled study clearly showed that a single intra-articular administration of allogeneic canine adipose derived MSCs improved the lameness score in dogs with chronic bilateral hip osteoarthritis. The effect was observed in some dogs as early as 5 days after IA administration of MSCs and it was prominent after 30 days of treatment and continued to improve lameness up to at least 90 days (as the last point of observation in this study). In addition to lameness scores performed by veterinarians, in this study we also collected information from pet owners about their pet's mobility and pain, utilizing the validated Canine Brief Pain and Inventory Score (CBPI). Data collected from the CBPI was in agreement with the veterinarian lameness score results, indicating that improved pet mobility, and reduced pain during daily activity as observed by the owner was consistent with improved lameness scores documented by the veterinarian. Due to a limitation in ground space in clinical trial sites, no objective lameness analysis method such as Gait analysis was used in this study. It should be noted that the dogs enrolled in this study did not receive any anti-inflammatory or pain reducing medications or any additional supplements or therapies at least 1 week before and during the 90-day study period.

Multiple factors can influence the outcome of the cell therapy including donor, recipient, dosing, cell therapy formulation, route of administration, and surgeon experience with joint injections. The quality and origin of the donor tissue has a great impact on the quality of cells obtained from that tissue. Previous studies showed that clinical improvements in signs of canine OA can be achieved using autologous stromal vascular fraction (SVF) from adipose tissue (43) or autologous adipose derived MSCs (44, 45). In our experience, derivation of MSCs from canine adipose tissue for autologous administration revealed that these cell products vary greatly between individuals, meaning that sufficient numbers of good quality SVF cells, and/or MSCs can only be obtained from a subset of patients (unpublished data). In this study, we used intra-abdominal adipose tissue from a young (5-months old) healthy canine donor to derive MSCs for allogeneic use. It has been demonstrated that with aging, the number of mesenchymal stem cells in the body diminishes (46) and this is the time that most of the patients develop osteoarthritis. Therefore, the availability of a good quality allogeneic mesenchymal stem cell product from a young healthy donor for these patients is essential.

The cell dose, number of injections, injection schedule, and site of administration also can change the outcome of the stem cell therapy. To date, limited information is available as to how many cells are required to exert a therapeutic effect on a patient with osteoarthritis. Different doses of MSCs were tested in this study. Interestingly all doses were effective in reducing pain and increasing mobility for a similar period. A phase I/II multicenter randomized placebo-controlled clinical trial with 30 human patients with knee OA revealed that administration of a higher dose (100 million) of autologous BM-MSCs has a more profound and sustainable effect than a lower dose (10 million cells) (47).

Most of the studies so far, including this study, used a single intra-articular dosing strategy. More studies are needed to investigate whether multiple administrations can improve the therapeutic effect. Repeated intra-articular administration of canine allogeneic adipose derived MSCs in healthy dogs is reported to be safe (48). We also found that a single intra-articular administration of allogeneic MSCs was well-tolerated, and except for transient joint pain in a few dogs, did not result in any severe adverse reactions. It has been reported that “joint flare” reactions can happen with intra-articular corticosteroid injections, and this may also be the case with administration of MSCs (49). Intra-articular administration of stem cells in patients with OA is the preferred method of administration because it concentrates the cells at the site of injury and potentially has a lower chance of distribution of stem cells to other organs. There is an ongoing debate about the contribution of MSCs to articular cartilage repair. While some investigators believe that MSCs disappear shortly after administration in the joint space (50), others have been able to locate the MSCs in articular cartilage up to a month after intra-articular injection (51).

In addition, the quality of the MSC manufacturing, experience level of the operators during preparation and administration of cells, the viability and functionality of cells after cryopreservation, the method in which the cells are prepared prior to patient administration (i.e., direct inject from thaw; thaw, wash and inject; culture recover then inject), and the adjuvant added to cells for resuspension and injection may all affect the effectiveness of cell therapy. Our MSCs were produced under FDA guidelines and consistently met rigorous specifications in order to ensure the highest quality. Cell surface marker expression, viability, proliferation rate, tri-lineage differentiation, MLR potency with increased PGE2 and IDO secretion and sterility, karyotype analysis and adventitious agent, and endotoxin screening were all performed. In addition, we used freshly thawed cells, washed away cryoprotectant, and resuspended them in saline for intra-articular administration. We used a controlled-rate freezer system for cryopreservation and a clinical grade cryoprotectant in our study and cells used in this study consistently had viabilities above 95% after thaw. We also found that the cell surface marker profile of the canine MSCs did not change after cryopreservation (data not shown). A recent study showed that intra-articular administration of freshly thawed allogeneic adipose derived MSCs in a DMSO-based cryopreservation medium was also effective in improving pain and lameness of dogs with osteoarthritis (38). While some investigators believe that MSCs need to be preconditioned (culture recovered) after cryopreservation and prior to administration for therapeutic use (52, 53), others believe that MSC functionality is not changed after cryopreservation (54, 55). Our data also clearly shows that freshly thawed and washed cells are functional as they improved lameness and pain in dogs with osteoarthritis. There are studies indicating that frozen-thawed MSCs may lose some of their immunomodulatory properties (52, 56). Further clinical studies directly comparing fresh thawed MSCs and fresh cultured MSCs need to be performed.

The extent of chronicity and severity of the degenerative joint disease in patients with OA can negatively impact the clinical outcome of a stem cell treatment protocol. Our study showed that younger patients had a similar response to MSC therapy suggesting that the age of the dog does not affect the response to MSC therapy. OA is more abundant in older age and our patient group also reflected that. While in our study the age of the dogs did not have an impact on the outcome of the stem cell therapy, a better response for younger dogs has been reported in a recent study using allogeneic adipose derived MSCs with a larger population of dogs with osteoarthritis (39).

Among many candidate biomarkers we tested in this study, two markers (IRAP and IL-10) showed an interesting relationship with lameness improvement. All the dogs in the Placebo group as well as the majority of the dogs treated with MSCs that also had improved lameness had an elevated blood IRAP 30 days after intra-articular administration. This indicates that elevated blood IRAP 30 days after intra-articular injection is a good indication of improvement of lameness irrespective to the treatment group. Increasing the blood level of IRAP in OA patients might be a natural response of the body to the arthritic condition. It has been well-documented that patients with OA have elevated levels of IRAP in their blood (57). The amount of IRAP in the systemic serum may be directly linked to the amount of lameness improvement. Chondroprotective effects of IRAP in delaying the progression of osteoarthritis in various experimental OA animal models including rabbit (58), dog (59), and equine (60) are reported.

Our data, for the first time, showed a positive correlation between the serum levels of IL-10 in dogs and their response to MSC administration. Interestingly, the level of IL-10 was maintained at a high level even 90 days after a single intra-articular dose of MSCs. More importantly, there was more IL-10 in the serum of dogs that received higher doses of MSCs, indicating that more stem cells resulted in a more profound IL-10 enhancement. How intra-articular administration of MSCs results in a higher elevation of IL-10 is unclear. Our MSCs produce IL-10 naturally, and thus they could release IL-10 in the synovial fluid and diffuse into peripheral blood following administration. Alternatively, MSCs can interact with host immune cells and induce IL-10 production. A stimulatory effect of MSCs on IL-10 secretion by the immune cells is reported (61).

In summary, this randomized, double-blinded, placebo-controlled pilot study clearly showed that a single intra-articular administration of canine allogeneic adipose-derived MSCs was well-tolerated and improved the lameness score and increased mobility of dogs suffering from hip osteoarthritis. As few as 5 million MSCs per joint were effective in reducing pain and increasing mobility. Lameness improvement was seen as early as 5 days after MSC administration and continued to improve during the course of the 90-day study period. Our data also provides some insight as to how MSCs may exert their anti-inflammatory effects in patients with osteoarthritis by systemic elevation of anti-inflammatory cytokines. While these results are encouraging, the small sample sizes and treatment groups composed of dogs with variable degrees of osteoarthritis and lameness remain as significant study limitations. Future studies should include more dogs and objective data collection for pre and post-injection gait analysis.

## Data Availability Statement

All datasets generated for this study are included in the article/Supplementary Material.

## Ethics Statement

Institutional Animal Care and Use Committee (ACUC) approval was not required because this study was conducted at private veterinary clinics using client owned dogs with owner consent. Also, approval was not required for this type of study under local legislation at the time. The dogs enrolled in this study were maintained in the owner's residence and were brought to the investigator sites for treatment and evaluation at the study's defined time points.

## Author Contributions

The authors on this paper qualify as have providing the following overall contributions: (1) substantial contributions to the conception or design of the work, or the acquisition, analysis, or interpretation of data for the work, (2) drafting the work or revising it critically for important intellectual content, (3) final approval of the version to be published, and (4) agreement to be accountable for all aspects of the work in ensuring that questions related to the accuracy or integrity of any part of the work are appropriately investigated and resolved. Specific contributions of each author were as follows: CM: study design and assembly, interpretation of data, evaluation of individual patients and clinical support, manuscript preparation, editing, and final approval. TR: manuscript review, administrative support, and final approval. RT, AB, C-BW, and JC all acted as study site investigators, provision of study material or patients and data collection. They also reviewed and approved the final version of the manuscript. DB: intellectual contribution in data interpretation, manuscript review, and final approval. FI: study design and assembly, data analysis, interpretation of data, editing/reviewing of manuscript drafts, and final approval of the manuscript. All authors contributed to the article and approved the submitted version.

## Conflict of Interest

The authors declare that this study received funding from VetCell Therapeutics USA. The funder had the following involvement with the study: providing the cell product, sponsoring the study and study design.

## Abbreviations

AD-MSC: adipose derived mesenchymal stem cells
BM-MSC: bone marrow derived mesenchymal stem cells
CBPI: canine brief pain inventory score
DMSO: dimethyl sulfoxide
ED: elbow dysplasia
ELISA: enzyme linked immunosorbent assay
HA: hyaluronic acid
HD: hip displasia
IDO: indoleamine 2,3-dioxygenase
IL-1 RA: interleukin 1 receptor antagonist
IL-10: interleukin 10
IRAP: interleukin 1 receptor antagonist protein
ISO: International Organization of Stan
LPS: lipopolysaccharide
MLR: mixed leukocyte reaction
MSC: mesenchymal stem cells
NSAID: non-steroidal anti-inflammatory drugs
OA: osteoarthritis
PGE2: prostaglandin E_2_
PRP: platelet rich plasma
PSGAG: polysulfated glycosaminoglycan
QC: quality control
SVF: stromal vascular fraction
VCT-USA: VetCell Therapeutics USA
VCT-Asia: VetCell Therapeutics Asia.
