# Supplementary material for: Intra-articular Administration of Allogeneic Adipose Derived MSCs Reduces Pain and Lameness in Dogs With Hip Osteoarthritis: A Double Blinded, Randomized, Placebo Controlled Pilot Study
Source: Front Vet Sci. 2020 Aug 31;7:570. doi: 10.3389/fvets.2020.00570 (PMC7489271; doi:10.3389/fvets.2020.00570)
Supplement: Supplementary file 1 [file Table_1.docx]

**Supplemental Table 1.** Lameness grading system used in this study [32, 33]

|  |
| --- |
| **Stance score R-HL_______, L-HL_______** |
| **0** Normal stance |
| **1** Slightly abnormal stance (favors limb but foot remains on floor) |
| **2** Severely abnormal stance (holds limb off of floor) |
| **3** Not able to stand |
|  |
| **Lameness at walk score R-HL_______, L-HL_______** |
| **0** No lameness and full weight bearing observed on all strides; normal gait |
| **1** Mild subtle lameness with partial weight bearing; dog may bear full weight on some strides and not others |
| **2**  Obvious lameness with partial weight bearing; dog is clearly lame on all strides |
| **3**  Obvious lameness with intermittent weight bearing; dog non-weight bearing on some strides, partial weight bearing on others. Includes dogs that “toe touch” on some strides |
| **4** Full non-weight bearing lameness; bears no weight on any strides |
|  |
| **Lameness at a trot score R-HL_______, L-HL_______. Does the pet trot?___________**  **(If pet does NOT trot, mention suspected reason for this _______________________________________________________________________________________________)** |
| **0** No lameness and full weight bearing observed on all strides; normal gait |
| **1** Mild subtle lameness with partial weight bearing; dog may bear full weight on some strides and not others |
| **2** Obvious lameness with partial weight bearing; dog is clearly lame on all strides |
| **3** Obvious lameness with intermittent weight bearing; dog non-weight bearing on some strides, partial weight bearing on others. Includes dogs that “toe touch” on some strides |
| **4**  Full non-weight bearing lameness; bears no weight on any strides |
|  |
| **Pain upon manipulation of affected joint through its normal range of motion score R-HL_______, L-HL_______** |
| **0** No pain elicited on palpation or movement of affected joint |
| **1**  Mild pain elicited (turns head in recognition) on palpation or movement of affected joint |
| **2**  Moderate pain elicited (pulls limb away) on palpation or movement of affected joint |
| **3** Severe pain elicited on palpation or movement of affected joint (vocalizes or becomes aggressive, or will not allow palpation or movement of affected joint) |
