# Supplementary material for: Intra-articular Administration of Allogeneic Adipose Derived MSCs Reduces Pain and Lameness in Dogs With Hip Osteoarthritis: A Double Blinded, Randomized, Placebo Controlled Pilot Study
Source: Front Vet Sci. 2020 Aug 31;7:570. doi: 10.3389/fvets.2020.00570 (PMC7489271; doi:10.3389/fvets.2020.00570)

**Supplemental Table 2:** Canine Brief Pain Inventory (CBPI) Assessment Form. (Reference copyright Dr. Dorothy Cimino Brown, University of Pennsylvania, <https://www.vet.upenn.edu/research/clinical-trials-vcic/our-services/pennchart/cbpi-tool>)


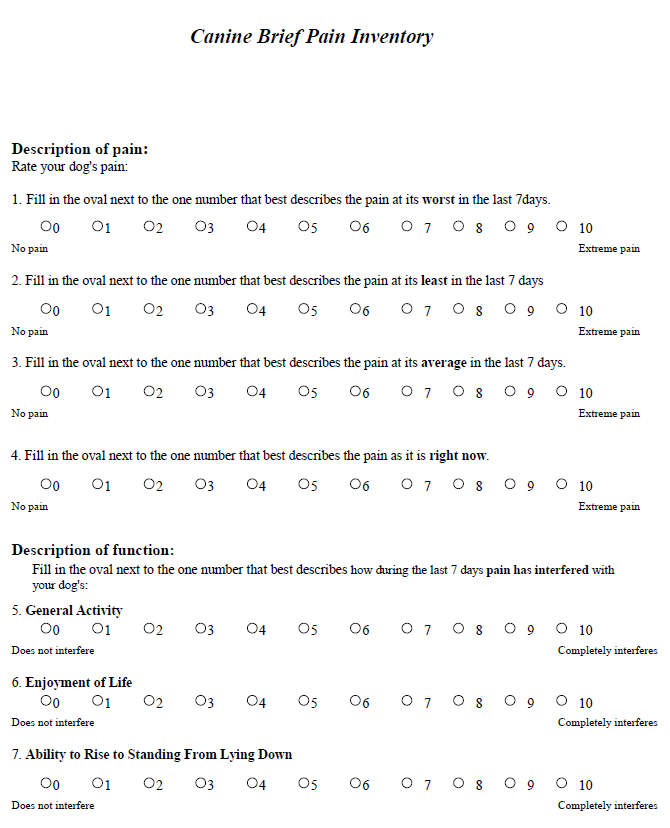


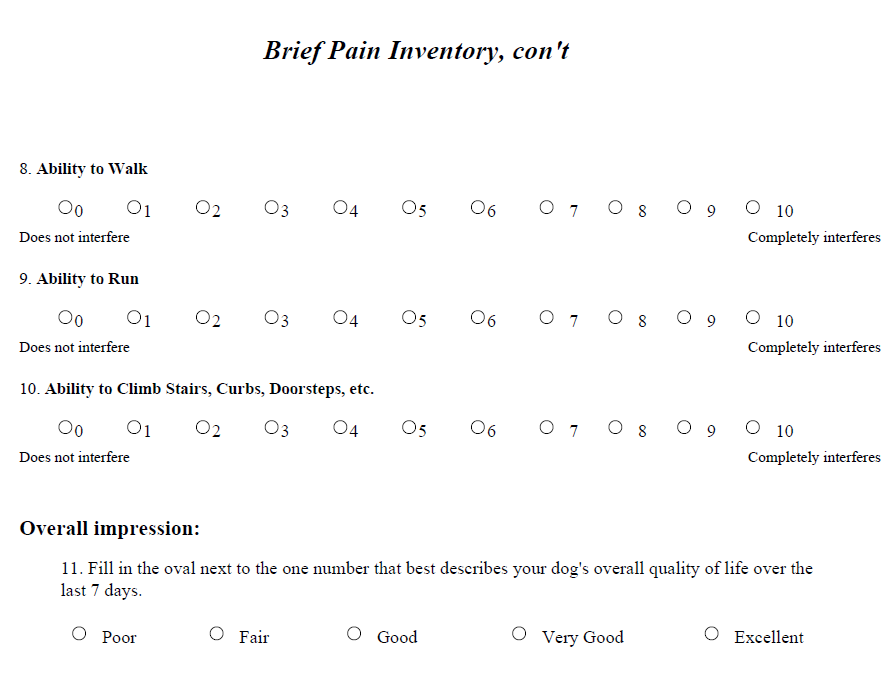

Supplement: Supplementary file 2 [file Table_2.docx]
