# Supplementary material for: Intra-articular Administration of Allogeneic Adipose Derived MSCs Reduces Pain and Lameness in Dogs With Hip Osteoarthritis: A Double Blinded, Randomized, Placebo Controlled Pilot Study
Source: Front Vet Sci. 2020 Aug 31;7:570. doi: 10.3389/fvets.2020.00570 (PMC7489271; doi:10.3389/fvets.2020.00570)
Supplement: Supplementary file 3 [file Table_3.pdf]

Supplemental Table 3. CBPI and Lameness scores of the individual dogs during the study

Treatment Group	Patient ID Number	Lameness Score			Pain Severity Reduction			Pain Interference Reduction			Limb Discomfort Post Injection (Y/N)
		Day 0	Day 30	Day 90	Day 0	Day 30	Day 90	Day 0	Day 30	Day 90	
Placebo	P-1	2	1	0	4	2	1.5	5	1.33	1.33	Y**
Placebo	P-2	1	0	2	0.5	0.25	0.25	1	0.17	0.17	N
Placebo	P-3	1	5	3	5	5	4.75	8.83	7.83	8.83	N
Placebo	P-4	5	5	4	5.8	6.1	6.2	6	6.17	6.17	N
5M	5-1	1	1	1	3	2.5	1.5	3.33	3.33	2.5	Y**
5M	5-2	2	1	0	4.25	4.75	4.7	5	4.5	4.67	N
5M	5-3	8	5	6	8	5	5.8	8.5	5	6.17	N
5M	5-4	8	5	5	7.6	5.7	5.3	8	5.67	5.5	N
5M	5-5	5	3	3	4	2.3	2.3	3.67	2	2	N
25M	25-1	1	3	NA	4	5	NA	9.17	5.3	NA	Y**
25M	25-2	1	0	1	0.25	0.75	1	1.17	0.83	0.83	N
25M	25-3	2	1	0	3	0.75	1.5	5.17	1.67	2.5	N
25M	25-4	5	2	1	2.5	1.5	1.3	3.83	2.5	1.5	N
25M	25-5	8	8	6	7	7.5	5.6	5.5	5.5	5.17	Y**
25M	25-6	7	7	7	5.4	5.4	5.4	4.83	4.83	4.83	N
50M	50-1	2	0	0	0.75	0.25	0.25	1.33	0.33	0.33	N
50M	50-2	1	0	0	3.25	3	2.2	7	4.5	2.17	N
50M	50-3	4	0	0	2.5	0.4	0	3.17	0.67	0	N
50M	50-4*	7	6	5	5.4	7.6	5.8	4.83	8	5.67	Y**
50M	50-5*	6	6	6	6.6	7.2	6.3	6.67	7.17	6	N

*: Two dogs were excluded from the study because they received other medications or treatments throughout the study that were not permitted in the inclusion criteria
**: Dogs with limb discomfort post injection
NA: Data was not collected
5M: 5 x 10^6 cells
25M: 25 x 10^6 cells
50M: 50 x 10^6 cells
